# Evaluation of realistic layouts for next generation on-scalp MEG: spatial information density maps

**DOI:** 10.1038/s41598-017-07046-6

**Published:** 2017-08-01

**Authors:** Bushra Riaz, Christoph Pfeiffer, Justin F. Schneiderman

**Affiliations:** 1 MedTech West and the Institute of Neuroscience and Physiology, Sahlgrenska Academy and University of Gothenburg, Gothenburg, Sweden; 2 Department of Microtechnology and Nanoscience – MC2, Chalmers University of Technology, Gothenburg, Sweden

## Abstract

While commercial magnetoencephalography (MEG) systems are the functional neuroimaging state-of-the-art in terms of spatio-temporal resolution, MEG sensors have not changed significantly since the 1990s. Interest in newer sensors that operate at less extreme temperatures, e.g., high critical temperature (high-*T*_c_) SQUIDs, optically-pumped magnetometers, etc., is growing because they enable significant reductions in head-to-sensor standoff (on-scalp MEG). Various metrics quantify the advantages of on-scalp MEG, but a single straightforward one is lacking. Previous works have furthermore been limited to arbitrary and/or unrealistic sensor layouts. We introduce spatial information density (SID) maps for quantitative and qualitative evaluations of sensor arrays. SID-maps present the spatial distribution of information a sensor array extracts from a source space while accounting for relevant source and sensor parameters. We use it in a systematic comparison of three practical on-scalp MEG sensor array layouts (based on high-*T*_c_ SQUIDs) and the standard Elekta Neuromag TRIUX magnetometer array. Results strengthen the case for on-scalp and specifically high-*T*_c_ SQUID-based MEG while providing a path for the practical design of future MEG systems. SID-maps are furthermore general to arbitrary magnetic sensor technologies and source spaces and can thus be used for design and evaluation of sensor arrays for magnetocardiography, magnetic particle imaging, etc.

## Introduction

Until recently low critical temperature superconducting quantum interference devices (low-*T*_c_ SQUIDs) were the only sensors that were commercially attractive for MEG systems because they uniquely combined high sensitivity with fabrication reproducibility. Recently, fabrication techniques have matured for high critical temperature (high-*T*_c_) SQUIDs with improved reproducibility^1, 2^ and sensitivity^3–5^ and new sensor technologies such as optically-pumped magnetometers (OPMs)^6^, diamond Nitrogen-vacancy center (N-V center) magnetometers^7^, giant magneto-resistance (GMR) based hybrid magnetometers^8, 9^, and kinetic inductance magnetometers (KIMs)^10^ have challenged the use of low-*T*_c_ SQUIDs in the next generation of MEG systems. Such new alternatives are attractive as they enable MEG with reduced head-to-sensor standoff (i.e., on-scalp MEG) because they require less extreme operating temperatures than conventional low-*T*_c_ SQUIDs (KIMs are the exception because they have yet to be demonstrated in high-*T*_c_ technology). While sensors developed in specialized labs have demonstrated improved noise levels compared to mass-produced low-*T*_c_ SQUIDs (c.f., e.g., refs 11–14), large-scale production of sensors that are suitable for multi-channel MEG systems are generally not as sensitive as low-*T*_c_ SQUIDs. However, that is partially compensated for by the gain in signal strength achieved by coming closer to the head surface. Some of these sensors have been used for proof-of-principle single- and multi-channel MEG recordings^15–25^. However, such recordings fail to demonstrate the neuroimaging advantage of these sensor technologies in full-head systems as compared to the state-of-the-art. An evaluation of the performance of new sensors in realistic full-head arrays is important both for demonstrating their advantages over today’s MEG, but also in the practical design of next-generation neuroimaging systems.

The development and optimization of modern MEG technology, from single-channel to full-head systems, was guided by theoretical studies comparing competing multichannel systems and optimizing the layouts of the low-*T*_c_ SQUID arrays^26–29^. With the emergence of improved sensor technologies, new simulation studies compared them with low-*T*_c_ based full head MEG systems with the aim of making a case for on-scalp MEG^30–32^. However, the sensor layouts suffered from being (i) arbitrary, e.g., a spiral array^32^, (ii) based on layouts that were optimized for conventional, rather than on-scalp, MEG sensor technology (i.e., snapping the sensor locations from current MEG systems to the head surface)^30, 31^ or (iii) impossible to implement from a practical point-of-view because the physical size of the sensor modules was not taken into account^31^. Realistic array designs for full head systems that exploit the full potential of on-scalp technology are thus lacking. Moreover, the layouts studied are mainly focused on designing a next generation MEG system with an adaptable array, comprising single moveable channels, such that the whole array fits snuggly on the head. The idea of a customized array for every individual head size is theoretically attractive, but performance would be limited by the size of a single sensor module in such an array. Larger modules will have sparse sampling/coverage of the brain. The more traditional helmet design, on the other hand, allows dense sampling by packing a high number of sensors in close proximity to one another. Finally, a compromise between fully flexible and a rigid helmet i.e., a modular approach consisting of many modules, each containing several tightly-packed sensors (distributed evenly to provide full-head coverage) has not been explored for new sensor technologies. As such, there is a lack of theoretical comparisons of practical approaches to on-scalp MEG systems with their state-of-the-art counterparts.

There is furthermore a lack of agreement on what metric should be used for evaluating the performance of sensor arrays in MEG. Total information capacity, *I*_*tot*_, has been suggested and used because it is simple and allows direct comparison of imaging systems as they sample neural activity in the brain while accounting for varying sensor array geometries, noise levels, and bandwidths^26, 29, 33^. It can furthermore potentially be used for other (neuro-) imaging modalities, e.g., fMRI, PET, etc. However, it fails to provide qualitative information, e.g., the spatial distribution of sensitivity. This is because *I*_*tot*_ is calculated based on a set of independent (orthogonalized) channels that are constructed with respect to how they sample the entire brain/source volume. As such, spatial information is lost in *I*_*tot*_ and coverage of specific brain regions cannot be assessed. More advanced simulation studies describe other spatial metrics, e.g., signal-to-noise ratio (SNR), relative sensitivity/signal power, and point-spread function (PSF) maps, to quantify the performance improvement on the cortical-level (c.f., e.g., refs 30, 31 and 34). While such methods enable comparison of the spatial distribution of an array’s sensitivity, they often rely on inverse operators that are strongly affected by the model of choice^31, 35^. Relative sensitivity maps allow comparison on the cortical level without the use of inverse operators. However, their use is limited because they quantify the relative coupling (interaction between sensors and sources) of the arrays by accounting only for the sensor geometry and locations. As such, these maps exclude sensor noise levels despite the critical role noise plays in MEG studies. SNR maps do account for sensor noise, but they do not cater for the redundant information inherent to MEG sensor arrays and can therefore be biased if, e.g., the numbers of sensors in two arrays that are being compared is not equal. What is therefore needed is a simple metric that accounts for sensor array geometries, noise levels, and bandwidths, provides spatial information (at the cortical level), does not rely on an inverse operator, and is unbiased by oversampling.

In this work, we present a comparison metric that combines information capacity with spatial information. It is based on the total information capacity that quantifies the information that can be extracted by a given sensor array from the entire brain (i.e., wherein all sources are active at the same time). However, we treat each cortical source individually and construct independent channels as the arrays sample each source. As such, we quantify the visibility of an individual source and give the maximum information that can be extracted when only said source is active in the brain. Our metric is in units of bits per source or, because MEG sources are cortical patches, bits per area and can be defined as spatial information density (SID). By treating the cortical sources independently, spatial information is preserved; SID-maps thus represent the spatial distribution of information density available to different MEG sensor arrays over the cortical surface. This metric therefore quantifies all aspects of sensor layout, sensor configuration, lead fields, noise levels, and distances from the sources without relying on inverse operators.

The SID-map furthermore serves as a design criteria for comparing—and optimizing—the design of MEG sensor arrays. We use it to assess three practical layouts for on-scalp MEG, based on high-*T*_c_ SQUIDs, in comparison to the standard Elekta Neuromag TRIUX magnetometer array. The three high-*T*_c_ on-scalp MEG system layouts we assessed are:

1. on-scalp MEG helmet, wherein sensors are tightly packed in a helmet shaped dewar. This layout is most similar to that which is standard in modern MEG systems; the major difference is that, because the sensors require less thermal insulation, the spacing between them and the head surface is significantly reduced.
2. single-channel on-scalp MEG, wherein the array consists of independently moveable (small) single-sensor cryostats surrounding the head. As such, it is fully flexible/adaptable to arbitrary head sizes and shapes, and
3. 7-channel on-scalp MEG, wherein the array consists of independently moveable (larger) cryostats (each of which houses 7 densely packed sensors) that surround the head. As such, it is semi-adaptable to arbitrary head sizes and shapes.

In order to compare and contrast the performance of each system for subjects with significant differences in head size and shape, we used two subjects for our simulations: a 35 year-old adult and a two year-old child. Sensor geometries, noise levels, and other parameters like sensor-to-sensor spacing, cryostat dimensions etc., for all on-scalp layouts are realistic estimates based on the literature and ongoing research in our lab. We furthermore evaluate the effects of the on-scalp/high-*T*_c_ SQUID sensor noise levels by using two values for each on-scalp layout: a conservative estimate of 50 fT/√Hz and a more optimistic 10 fT/√Hz.

## Results

### Total information capacity

**Figure 1:**
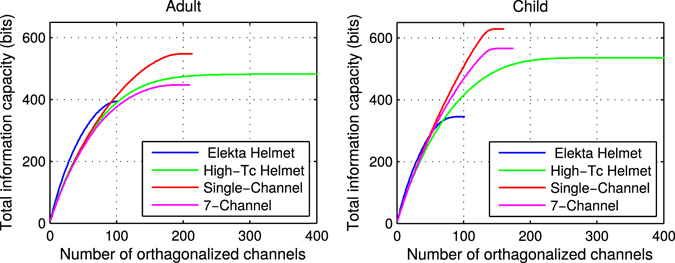
Cumulative sum of information capacity for Elekta and high-*T*_c_ SQUID arrays as they sample the adult (left) and child (right) brains. The high-*T*_c_ SQUID noise level used here is conservative (50 fT/√Hz) whereas the Elekta SQUID noise is 3 fT/√Hz. Only the first 400 orthogonal channels are presented because the cumulative information does not increase significantly beyond that. In the adult case, the slope for the Elekta system is higher than all high-*T*_c_ arrays. In the child case, the upward trend is similar for all arrays. In all cases, the total information available to the high-*T*_c_ arrays is higher than the Elekta system.

A sample of the total information accumulated from orthogonalized channels (Material and Methods, equation 1) for the Elekta and high-*T*_c_ SQUID layouts (for adult and child) are shown in Fig. 1. We present only the conservative case of 50 fT/√Hz high-*T*_c_ noise levels in order to illustrate trends in accumulated information and enable straightforward comparison to previous work (for optimistic case of 10 fT/√Hz high-*T*_c_ noise level, see Supplementary Fig. S1). All of the relatively noisy high-*T*_c_ SQUID-based MEG arrays extract more total information from both adult and child brains as compared to the state-of-the-art MEG systems.

The total information, *I*_*tot*_, for all MEG arrays and both subjects is presented in Table 1.

**Table 1:** Total information capacity in bits per sample for each sensor array and subject.

	Elekta Neuromag Helmet	High-*T*_c_ on-scalp MEG helmet		Single-Channel on-scalp MEG		7-Channel on-scalp MEG	
	σ_Noise,L***T*c**_ = 3 fT/√Hz	σ_Noise,H***T*c**_ = 50 fT/√Hz	σ_Noise,H***T*c**_ = 10 fT/√Hz	σ_Noise,H***T*c**_ = 50 fT/√Hz	σ_Noise,H***T*c**_ = 10 fT/√Hz	σ_Noise,H***T*c**_ = 50 fT/√Hz	σ_Noise,H***T*c**_ = 10 fT/√Hz
Adult	393	482	999	547	984	447	845
Child	345	535	1054	629	968	566	909

With the improved noise level of 10 fT/√Hz, the high-*T*_c_ SQUID arrays extract at least twice as much information from the adult brain and 2.5 times more information from the child brain as the Elekta system. Furthermore, while the Elekta system extracts less total information from the child as compared to the adult brain, all high-*T*_c_ SQUID arrays, with the exception of the low-noise single-channel on-scalp system, extract more information from the child brain as compared to the adult.

**Figure 2:**
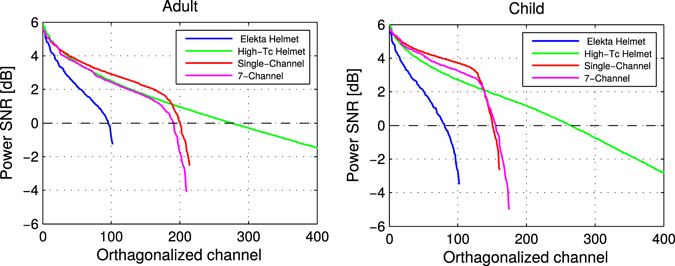
Power SNRs for all MEG arrays over orthogonalized channels for an adult (left) and a child (right) brain with optimistic high-*T*_c_ SQUID noise levels (10 fT/√Hz). The zero crossing indicates the number of channels that achieve SNR > 1.

Comparison within the three high-*T*_c_ layouts shows that the information extracted from all three layouts is not significantly different (within 20%), even though the number of sensors in the helmet design varies significantly. The trend in accumulated information across channels in Fig. 1 also indicates that not all of the orthogonalized channels are adding to the information content; that is especially the case for the high-*T*_c_ helmet array. This aspect can be understood via inspection of the power signal-to-noise ratio (P_k_ values from Material and Methods equation 5) of the orthogonalized channels, plotted in Fig. 2.

In the case of the Elekta helmet, the single-, and 7-channel high-*T*_c_ SQUID systems, the SNR of the last 10 to 20 orthogonalized channels falls below 0 dB i.e., the SNR is 1 or lower. As such, additional orthogonalized channels do not significantly add to the total information. For example, only the first 259 orthogonalized channels in the high-*T*_c_ helmet design have an SNR of greater than one.

### SID

**Figure 3:**
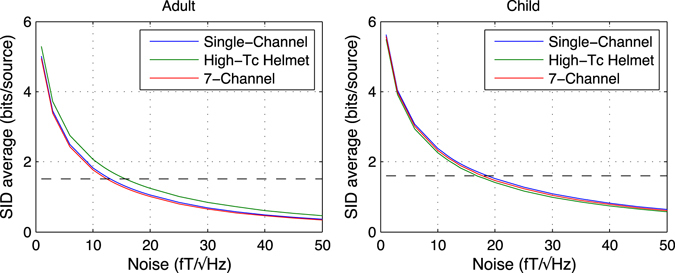
*SID*_*avg*_ for three on-scalp arrays as a function of sensor noise levels for recordings on the adult (left) and child (right). The dashed, horizontal line is *SID*_*avg*_ for the Elekta helmet array (with sensor noise levels of 3 fT/√Hz) for comparison (1.5 bits/source for the adult, 1.6 bits/source for the child). Intersections between the on-scalp *SID*_*avg*_ lines and this reference line indicate noise levels required to reach the performance of the Elekta system with respect to average sampling of independent sources.

In Fig. 3, we present the average spatial information density, *SID*_*avg*_, for the three high-*T*_c_ SQUID sensor layouts as a function of sensor noise from 1 to 50 fT/√Hz. The *SID*_*avg*_ for the Elekta system (1.5 and 1.6 bits/source for the adult and child, respectively, with fixed 3 fT/√Hz noise levels) is included as a dashed horizontal line for reference. For optimistic noise levels in high-*T*_c_ SQUID sensor technologies of 10 fT/√Hz, *SID*_*avg*_ is better than that of the Elekta system for both the adult and child cases. In the adult case, the high-*T*_c_ helmet array gives the highest *SID*_*avg*_, extracting 40% more information per source as compared to the Elekta array. By contrast, the single-channel array performs best for the child case, extracting on average 50% more information per source than the low-*T*_c_ SQUID array. However, for high-*T*_c_ SQUID sensors with more conservative noise levels of 50 fT/√Hz, *SID*_*avg*_ is far lower. As such, it is most illustrative to present SID maps for each of the of high-*T*_c_ SQUID layouts for the optimistic high-*T*_c_ SQUID noise levels in parallel with that of the Elekta system because they can all be presented with the same scale i.e., the variation between SID maps can be compared directly.

**Figure 4:**
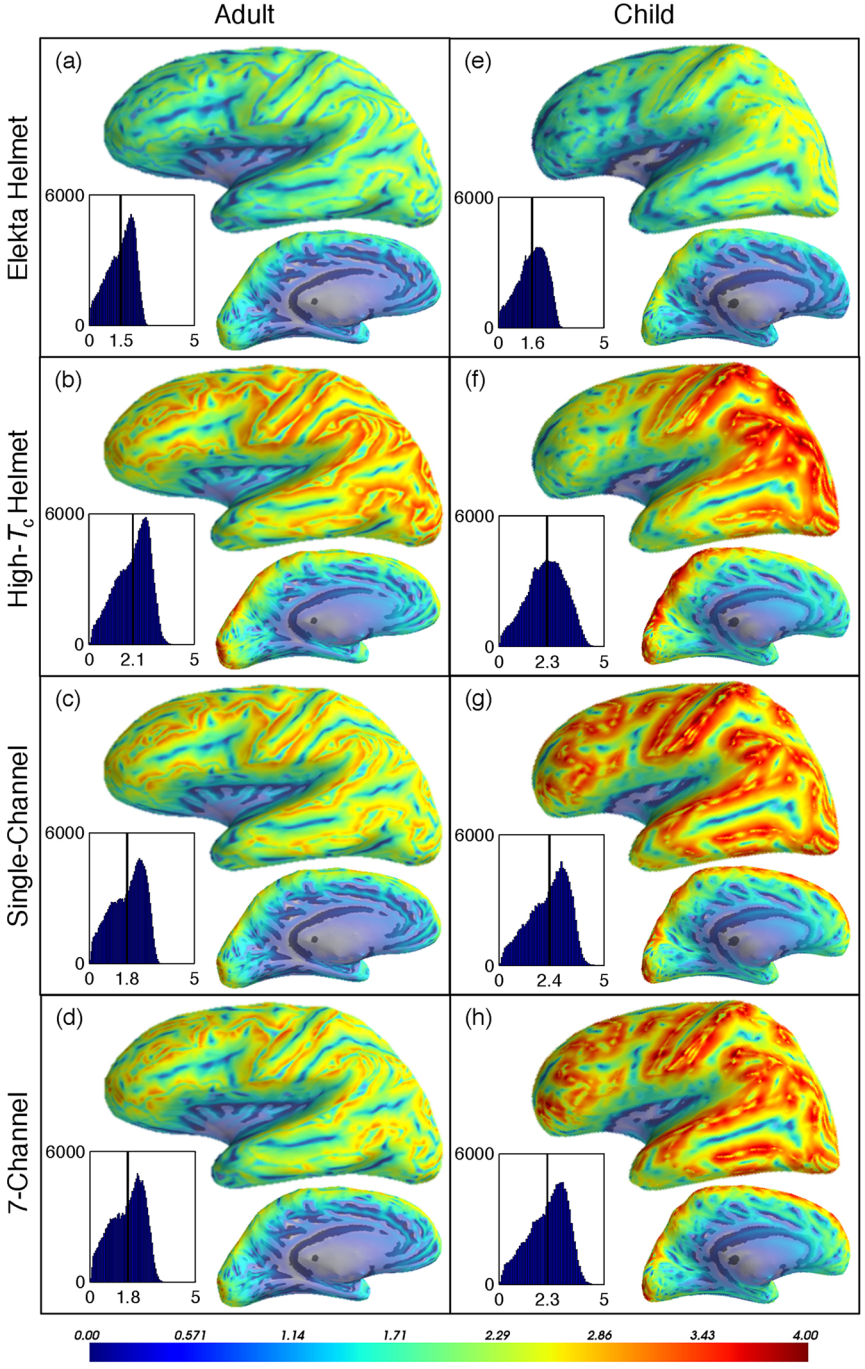
SID-maps for all layouts sampling the adult and child brains (left and right columns, respectively). Each panel displays lateral and medial views of the left hemisphere of the brains; results do not differ significantly for the right hemisphere. We use optimistic noise levels for the on-scalp sensors (10 fT/√Hz). Histograms of the distribution of SID values for both hemispheres are included (with a vertical reference line marking *SID*_*avg*_ for each layout-subject pair). Coverage is roughly uniform for all on-scalp layouts for both subjects. A weak gradient from the frontal to occipital lobes for the adult brain as sampled with helmet layouts is far more pronounced for the child. Peak and average SID values for the on-scalp layouts recording from the child brain are significantly higher than for the adult.

SID maps for all MEG arrays for both subjects are shown in Fig. 4. The color map represents the number of bits extracted at each source location.

**Figure 5:**
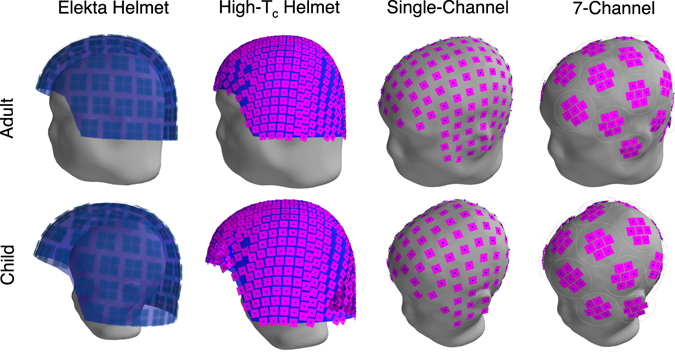
The sensor positions (pink with a blue dot in the center) in all layouts for sampling the child and adult brains are shown. The helmet surfaces in the Elekta and high-*T*_c_ helmet layouts are shown in blue.

The SID maps for the helmet-based arrays (Fig. 4 panels a, b, e and f) demonstrate the limitations of the conventional “one size fits all” approach for MEG. There is a gradient in the SID from front to back of both the adult and child brains for both helmet arrays. In each case, the subject’s head is positioned as it would be in a typical MEG recording: with the back part of the head in contact with the back of the helmet and the top part of the head as high as possible (Material and Methods and Fig. 5). While the sensors are well positioned/close to the top and posterior parts of the brain, they are far from the front. This gradient is more pronounced for the child brain because of its relatively small head as compared to the adult. However, the posterior part of the child brain has higher SID values as compared to the adult for both helmet arrays. This is because the minimum cortex-to-scalp distance for the child (4.4 mm) is smaller than the adult’s (9.6 mm) and the posterior part of the child’s brain is therefore in closer proximity to the sensors in the back of both helmets.

Comparing SID maps for the single- and 7-channel high-*T*_c_ SQUID arrays provides guidance for cryostat design that goes beyond the traditional helmet approach. Despite the differences in uniformity of sensor distribution around the head, these quite different layouts both provide uniform coverage for both subjects throughout the cortex. However, the temporal lobe is best sampled with the single-channel array. Furthermore, the 7-channel array extracts less information per source (averaged for all sources) compared to the single-channel array.

It is also worth noting that the overall SID values are higher for the child as compared to the adult. This is likely to be because a child’s brain is closer to the scalp surface than an adult’s and the sensors are therefore, on average, closer to the child’s brain. As such, all on-scalp arrays provide significantly better coupling between the sensors and the child’s brain as compared to the adult’s.

## Discussion

The aim of this study was to assess three practical layouts for on-scalp MEG with a traditional (total information capacity) and a non-standard, but potentially advantageous, comparison metric - SID-maps. The layouts were based on realistic design parameters for high-*T*_c_ SQUIDs. We analyzed performance for MEG recordings on two subjects with different head sizes, a 35-year-old adult and a two-year-old child. The inclusion of the child in our study enabled us to explore the benefits of on-scalp MEG for subjects with smaller-than-average head size.

All high-*T*_c_ SQUID on-scalp arrays outperformed the low-*T*_c_ Elekta Neuromag array in terms of total information extracted, even with conservative high-*T*_c_ noise levels (50 fT/√Hz). The single-channel on-scalp design gives the highest, whereas the 7-Channel on-scalp array gives the lowest, *I*_*tot*_ for both child and adult with such relatively noisy sensors. However, the differences are minor (within 20% of each other): the total information capacity metric thus provides only general evidence that on-scalp MEG is a viable approach. Worth noting, though, is that the relative gain in information per sample for all high-*T*_c_ arrays (as compared to the Elekta system) is higher for recordings on the child than with the adult, showing the improved potential of on-scalp MEG for studies on children.

*I*_*tot*_ also provides relevant information with regards to system design vs. sensor noise levels. For example, while the single-channel on-scalp MEG layout yields more information for conservative sensor noise levels, the helmet array provides more information for optimistic noise levels. This is likely because noisier sensors that are also far from the head in the helmet layout fail to add information; this is apparent in the slow rise of the information capacity for the helmet layout with relatively noisy sensors in Fig. 1. However, when the sensor noise is improved from 50 to 10 fT/√Hz, sensors in the helmet that are far from the head presumably add considerable information (as can be seen in the long tail of the SNR values for the low-noise helmet array in Fig. 2). The helmet layout thereby yields more total information than its single-channel counterpart. As such, according to *I*_*tot*_, noisier sensors benefit from closer proximity (where packing is necessarily less dense due to practical limitations) whereas low-noise sensors benefit from being packed more densely (despite the resulting practical cost of being farther from the scalp surface).

Our simulations validate previously reported findings. For example, the single-channel on-scalp MEG array with 50 fT/√Hz noise extracts 39% more information from the adult brain. These results are comparable to previous findings where high-*T*_c_ SQUIDs with the same noise levels extracted 40% more information compared to a low-*T*_c_ SQUID array, despite the use of a crude spherical head model and simple spiral layouts for the sensor arrays^32^. The reported results are also in agreement with a more recent study in which another sensor technology for on-scalp MEG - OPMs - are evaluated. Iivanainen *et al*. simulated an array of 102 OPMs with sensor noise levels of 6 fT/√Hz vs. the Elekta magnetometer array. The OPM system yielded 656 bits per sample (vs. 413 for the Elekta case), averaged for 10 adult head/brain models^30^. Such numbers are in very close agreement with those presented herein: 102 of the orthogonalized channels in the single-channel high-*T*_c_ on-scalp MEG array (with noise levels of 10 fT/√Hz) accumulate 653 bits (vs. 393 for the Elekta case).

The information content results enable quantification of the general performance of the sensor arrays; however, the coupling and the sensitivity of the arrays to different parts of the brain cannot be resolved with such a simple metric. As indicated above, one can only speculate reasons for differences in information capacity, especially when comparing the on-scalp systems to one another. While the information content from on-scalp arrays is comparable for each layout, the arrangement of the sensors on the scalp is quite different. It is therefore illuminating to use SID maps for presenting information content at the cortical level. Questions regarding the visibility of specific brain regions, sensitivity to deep sources, and even how to optimize layouts can therefore be answered. Moreover, this spatial metric is acquired without solving the inverse problem and thus avoids possible bias generated by inverse method selection. The SID values obtained on the cortical surface quantify the amount of information the sensor array can extract from each individual.

The average of SID values over the cortical surface/source space, *SID*_*avg*_, is an additional metric that supplements *I*_*tot*_. For example, we use it to estimate the sensor noise level required by a particular layout in order to achieve the same, or better, coupling to the entire set of independent cortical sources as compared to low-*T*_c_ SQUID systems. Interestingly, while *I*_*tot*_ for all on-scalp systems with conservative sensor noise levels (50 fT/√Hz) is higher than that of the Elekta system, *SID*_*avg*_ is considerably lower. This is because *I*_*tot*_ is based on summation of all signals generated in the brain whereas *SID*_*avg*_ is based on coupling to individual - and independent - sources. The overall signal magnitude generated by the entire brain is therefore high compared to the sensor noise levels, and *I*_*tot*_ is subsequently boosted. On the other hand, individual sources that are treated independently are weak/lower in magnitude compared to the sensor noise levels, SNRs are then lower, and so is *SID*_*avg*_. Figure 3 indicates on-scalp high-*T*_c_ SQUID noise levels should be around 15 fT/√Hz (Adult) and 18 fT/√Hz (Child) to perform on par with the Elekta system for MEG recordings on an adult and child, respectively. As with *I*_*tot*_, the differences between the layouts as quantified with *SID*_*avg*_ are marginal: the layouts perform nearly identically in the child case, whereas the helmet array is slightly better than the others for the adult.

The *SID*_*avg*_ values vs. sensor noise in Fig. 3 indicate it is reasonable to compare SID-maps for all of the on-scalp arrays with the same scales as that of the Elekta system with our optimistic high-*T*_c_ SQUID sensor noise level of 10 fT/√Hz. Those SID-maps are presented in Fig. 4 and allow us to both quantitatively and qualitatively describe and compare each of the on-scalp MEG layouts to one another and the Elekta system as follows.

### Helmet arrays

The SID-maps reveal a lack of uniformity in sensitivity to the cortex with both the on-scalp and Elekta helmet designs for both the adult and child (Fig. 4 panels a, b, e, and f): in all cases, the frontal lobes have lower SID than occipital. However, the gradient is weaker for the adult cortex, as expected, because the adult head fits relatively well in the helmet. For children, the non-uniformity in SID with the helmet is far more significant. Frontal areas are sampled poorly, whereas occipital areas (where sensors are densely packed and very close to the brain) have far better coverage/higher SID values than all other helmet-subject combinations. These results suggest having multiple helmet sizes that better match individual head sizes is a worthy approach, especially for MEG studies on children^36^ (this has been implemented with low-*T*_c_ sensors, e.g., Infant MEG system -MagView^37^, Tristan Technologies and Compumedics/KRISS dual MEG^38^).

The total number of sensors in the on-scalp helmet array is almost 3 times higher than in the other on-scalp arrays. However, information and SNR plots in Figs 1 and 2 show that only around 250 orthogonalized channels are adding independent information. Even though spatial oversampling is always needed, the saturation seen already after 250 orthogonalized channels indicates unnecessary oversampling by physical sensors. This suggests the helmet array could be more efficiently designed, e.g., by including fewer sensors that are packed less densely. This would also provide space for increasing the pickup loop size of the sensor, thus improving their sensitivity^39^. Such design parameters can be optimized for *I*_*tot*_ and/or *SID*_*avg*_ while using the SID-map to ensure uniform coverage of the whole cortex.

With the development of new sensor technologies, the idea of having all of the sensors on the scalp surface is of increasing interest. However, our results indicate traditional helmet designs may still be preferable even for these new sensors. Such a layout is furthermore more straightforward to construct and benefits from being the standard in MEG. As such, co-registering/tracking the sensor array position relative to the head/brain, forward modeling and inverse methods^31^, etc. are straightforward to implement based on well-established solutions. In the helmet design sensor positions and orientations with respect to each other are calibrated and stable, whereas a flexible design requires re-defining the layout on a subject-by-subject basis. Moreover, keeping parts of a flexible array fixed with respect to one another over the course of a recording is also a challenge.

### Single-channel on-scalp array

This layout is based on flexible hardware with a single, independently moveable cooling unit per SQUID. The SID-maps indicate it provides uniform coverage for both the child and the adult because the sensors are all within a few mm of the head surface. This layout furthermore produces the highest *SID*_*avg*_ values for the MEG recordings on the child, at least partially because of the relatively small scalp-to-brain distance^40^. Also of interest: the performance of the 150+ sensors in the single-channel layout is comparable to (in some cases even better than) the 600+ sensors in the helmet one.

The single-channel solution seems very attractive in terms of enhanced and uniform performance irrespective of head size while reducing the number of sensors per full-head system. However, this layout also entails additional complications, e.g., more complicated forward modeling is required. With a single-channel design, each subject would have a customized array layout that requires new methods for co-registering individual sensor locations to head positions. 3D-printed head cast solutions have already demonstrated potential in conventional helmet-based systems^25, 41, 42^ and might be adaptable for single-channel systems. However, this adds to overheads in terms of preparation cost and time while increasing subject discomfort. Moreover, head casts are meant to restrict head movement and would therefore not be suitable for infants and children, as they tend to have less tolerance for such restrictions.

The packing density of single-channel on-scalp cryostats - and therefore the performance of this layout - is limited by the thickness of the cryostat walls. Commercially available micro-cryocooling technology enables significant reduction in the cold volume of the system; higher packing density should then be possible^43–45^ (c.f., Kryoz Technologies BV). The footprint of micro-OPM and diamond NV-center magnetometers could be well suited for single-channel on-scalp layouts^7, 46^. However, with the current constrains of cryostat wall thickness, the single-channel on-scalp system performance is comparable to the helmet design for the adult case.

### 7-channel on-scalp array

The 7-channel on-scalp array was evaluated as a compromise between flexibility and packing density. Because it is a more bulky cooling approach than the single-channel coolers, gaps between cryostats are expected to lead to patchy SID-maps. Interestingly, the SID maps show that the coverage from this layout is uniform and does not differ significantly from the single-channel array. Like with the single-channel system, far fewer sensors are required for equivalent performance as compared to the helmet approach. Packing more than seven channels in a single unit and constructing the cryostats with varying shapes and curvature to match different parts of the scalp can potentially lead to better performance than the helmet and single-channel layouts. Furthermore, while the modular approach still imposes co-registration and forward modeling problems, the negative effects could be mitigated more easily than with a single-channel on-scalp layout, e.g., because the solution for localizing a set of sensors (whose relative positions with respect to one another is well known) with a set of head position indicator coils (as is standard in conventional MEG) is far better determined than in the case of a single sensor.

## Conclusions

We have assessed and compared three practical on-scalp MEG sensor layouts with the conventional Elekta Neuromag system. Results with a conventional measure, namely total information capacity, match previous reports and suggest on-scalp MEG is worthy of further pursuit. Using total information capacity, high-*T*_c_ SQUID arrays with even a conservative sensor noise estimate of 50 fT/√Hz are better than a standard low-*T*_c_ Elekta Neuromag system with 3 fT/√Hz sensor noise. The same metric also indicates an on-scalp array consisting of 214 sensors (161 for the child array) outperforms a helmet array with 631 sensors when the noise level is conservative, but not when it is optimistic. The SID maps provide complementary and unique information, suggesting, for example, that helmet-shaped dewars are preferred for subjects with average head sizes or if uniformity of sensitivity is not necessary (e.g., because a specific region of interest has been identified or multiple recordings with different head-to-helmet alignments is tolerable). For subjects with smaller and/or unusually-shaped heads, more flexible on-scalp systems significantly improve sensitivity to the entire cortex.

## Material and Methods

### Anatomical Model

In this simulation study we used T1-weighted MRIs of 2 subjects: a 35 year-old male and a 2 year-old child (the latter of which was obtained from the open repository brain-development.org^47^). The adult subject gave written consent for the use of his MR image that was obtained as a part of a study carried out according to approval by the Gothenburg Regional Ethical Review Board (dnr. 488-12) in accordance with the declaration of Helsinki. The span of subject ages was selected in order to enable comparison of the performance of the arrays for heads of very different size. FreeSurfer^48–50^ was used to segment the MRIs. The source space was generated by the standard approach of distributing dipoles on the mid surface between grey and white matter surfaces with approximate source-to-source spacing of 2 mm. Because the adult’s cortical surface area (94 030 mm^2^) is larger than that of the child (77 637 mm^2^), the number of sources in the adult source space (156 176) significantly exceeds that of the child (127 931). For dipole strength we use values reported in the literature: studies have shown that the maximum current density on the cortical surface is relatively constant across brain structures. For the human neocortex the values reported are in the range of 0.16–0.77 nAm/mm^2^ ^51^. Adding this physiological constraint in inverse modeling has improved the estimation of the active regions based on minimum norm estimates^52^. Measures like information capacity and SID values, where we are not only interested in relative comparison with the current system but also the absolute values from individual layouts, benefit from such plausible physiological constraints on current density. High source amplitude for simulations artificially benefits all systems, especially those with noisier sensors. Specifically, high source strength values result in an artificial lack of saturation in the information capacity plots and SNR is likewise shifted to unrealistically high values. We have furthermore chosen equidistance spacing of dipoles on the cortical mantle. As such, each dipole represents a fixed patch of activated cortex and is thus assigned signal strength accordingly (based on the current density reported in the literature). In our simulations with 2 mm source spacing, the average cortical source patch size is 0.6 mm^2^, the current density is fixed at 0.7 nAm/mm^2^ (i.e., within the range, but below the maximum, of reported physiological values) and the resulting dipole strength per source patch is 0.4 nAm (for both the adult and child).

### Sensor Specification

We compared magnetometers in both low- and high-*T*_c_ sensor technologies^32^. We employed the MNE-generated model of the Elekta Neuromag low-*T*_c_ SQUID sensor array^53^. The pickup loop size was 25.8 mm × 25.8 mm with noise levels of 3 fT/√Hz. The pickup loop for all high*-T*_c_ SQUID sensors used in these simulations was 8 mm × 8 mm as this is a typical size for readily available 10 mm × 10 mm substrates. We compared two different noise levels for high*-T*_c_ SQUIDs with such size limitations: a conservative (50 fT/√Hz) and an optimistic one (10 fT/√Hz). Sensors of this size typically reach white noise levels below 50 fT/√Hz down to 1 Hz^16, 18^. The optimistic noise level of 10 fT/√Hz was based on work by Faley *et al*.^12, 20^, but is considered optimistic because fabrication of such low-noise high*-T*_c_ SQUIDs is more complicated (i.e., it requires multilayer high*-T*_c_ technology that has only been demonstrated by a limited number of labs) than the more traditional bicrystal technology^54, 55^.

### Layout of the sensor arrays with respect to the subjects’ heads

#### Elekta Neuromag Helmet

The low-*T*_c_ SQUID array was positioned such that both subjects were resting their heads on the back of the helmet as shown in Fig. 5. The minimum sensor to room temperature distance in the Elekta Neuromag dewar is approximately 20 mm. The closest sensors from the scalp surface (i.e., those at the back of the head) were 21 mm away for both the adult and child head models.

#### High-*T*_c_ MEG helmet

In this design a large number (631) of high-*T*_c_ SQUIDs were tightly packed in a single helmet-shaped dewar similar to the one used in the Elekta system. Increasing the density of sensor packing can result in issues related to crosstalk between adjacent sensors. This crosstalk is manifested via mutual inductance between the SQUID feedback signals. However, the crosstalk between high-*T*_c_ sensors can be reduced to less than 0.5% via direct injection of the feedback current into the SQUID loop, even when the spacing between neighboring sensors is reduced to 1 mm^56^. The helmet design is advantageous with regard to the density of sensor packing as all sensors share the same thermal insulation. The dewar wall and layer of thermal isolation for this layout was 1 mm thick. While such a thin wall and insulation layer is a practical challenge in a helmet design, it was chosen so that the sensor-to-room-temperature distance would be on par with that of the other on-scalp layouts.

The high-*T*_c_ sensors were arranged on the helmet surface with a semi-automatic procedure in which a square grid of equidistance points is generated using an automated Chebyshev net algorithm^57^. Nodes along the two primary axes of the grid were separated by at least 12 mm. Such a design allowed 631 high-*T*_c_ sensors to be distributed on the helmet surface. The relative position of the subject’s heads with respect to the helmet was the same as that of the Elekta Neuromag helmet, i.e., the subjects were resting their heads at the back of the helmet. The closest sensors (as in the Elekta layout, those that are at the back of the head) were at least 1 mm away from scalp surface.

#### Single-channel on-scalp MEG

In the single-channel on-scalp layout, full head coverage was obtained by aligning a large number of cryostats, each of which houses a single high-*T*_c_ SQUID, to the head surface. The layout was generated on the head surface with the same Chebyshev net algorithm as with the high-*T*_c_ SQUID helmet and a center-to-center spacing of at least 20 mm. This relatively high i.e., conservative, spacing accommodates realistic cryostat bodies for independently cooling each sensor. Each cryostat is assumed to require 5 mm (e.g., 1 mm vacuum +4 mm support/wall) of sensor-to-room-temperature spacing around the edges of the SQUIDs in order to accommodate the tight (~1 mm) sensor-to-room-temperature spacing over the SQUID chip’s 10 mm × 10 mm surface. All sensors were at least 1 mm away from the scalp surface. For such an on-scalp array of single-channel cooling systems, the total number of sensors for the adult and child was 214 and 163, respectively.

#### 7-channel on-scalp MEG

In the 7-channel on-scalp layout seven sensors, arranged in a hexagonal pattern, are housed in one cryostat similar to a system under development in our lab. Several such cryostats are aligned to the head surface to enable flexible full-head coverage. The sensors within a cryostat are tilted towards the center to roughly match the average radius of curvature of the head (80 mm). The radius of the sensor holder is 21 mm; the vacuum gap (2 mm) and cryostat wall (4 mm) that surround the holder then yield a center-to-center cryostat spacing of at least 54 mm. The layout for these cryostats was generated manually via arranging the cryostats as densely as possible on the subject’s heads. All sensors were at least 1 mm away from the scalp surface. 25 and 30 of these 7-channel cryostats were distributed on the head surface for the child and adult cases, respectively.

Array parameters are summarized in Table 2.

**Table 2:** Geometric parameters for sensor array layouts.

	Average sensor standoff from scalp surface (mm) *Adult*/*Child*	Total number of sensor *Adult*/*Child*	Sensor spacing (center-to-center)
Elekta Neuromag Helmet	28.9/40.2	102/102	34 mm
High-*T*_c_ MEG helmet	9.6/21.2	631/631	12 mm
Single-channel on-scalp MEG	1/1	214/161	20 mm
7-channel on-scalp MEG	1.9/1.8	210/175	12 mm*

*Sensor spacing within a single cryostat. Inter-cryostat spacing was 54 mm.

### Forward Model

The forward solution/gain matrix was calculated using a single layer linear collocation boundary element model (LC BEM) implemented in MNE^53, 58^. The brain compartment was segmented using the watershed algorithm in FreeSurfer and 0.33 S/m conductivity was assigned to it. The BEM mesh for the brain compartment consisted of 2562 vertices. Iivanainen *et al*.^30^ have shown that the use of more accurate BEM solvers such as linear Galerkin and generation of a BEM mesh with more than 2562 vertices have no significant improvement in forward computation of on-scalp magnetometers as compared to low-*T*_c_ SQUIDs. The dipole orientations were fixed normal to the cortical mantle. The output of the sensors was calculated by integrating the magnetic flux over a grid of 16 points evenly distributed within the pickup coil loops for both high- and low-*T*_c_ SQUID sensors.

### Information Content

The information content in each MEG array is calculated based on Shannon’s theory of communication^59^ and the calculations proposed by Kemppainen and Ilmoniemi^26^

1 $${I}_{tot}=\frac{1}{2}\sum _{k}{\mathrm{log}}_{2}({P}_{k}+1)$$

where *P*_*k*_ is the power signal-to-noise ratio (SNR) of the k-th orthogonalized channel of the array and *I*_*tot*_ is the total information from the entire array as it samples the whole source volume. For summing the power over individual channels, the information in each channel must be independent. This can be achieved by orthogonalizing the sensors’ lead fields.

With the assumption that primary currents obey a Gaussian distribution, $J \sim N(0,{\sigma }_{signal}^{2})$, the power SNR for a channel with noise N is given as

2 $$P=\frac{{S}^{2}}{{N}^{2}}=\frac{{\sigma }_{signal}^{2}}{{N}^{2}}{(\int |\overrightarrow{L}(r)|dr)}^{2}$$

where $|\overrightarrow{L}(r)|$ is the magnitude of the lead field.

The inner product of the lead field matrix Γ is calculated from the forward solution/gain matrix G as follows^60^

3 $${\rm{\Gamma }}={\int }_{V}\overrightarrow{{L}_{i}}\,\overrightarrow{{L}_{j}}dv=G{G}^{T}$$

where $\overrightarrow{{L}_{i}}$ and $\overrightarrow{{L}_{j}}$ are the vector lead fields of sensors i and j. In order to orthogonalize the channels, Γ is decomposed via singular value decomposition (SVD) to:

4 $${\rm{\Gamma }}=U\lambda {U}^{T}$$

where U is a matrix containing the eigenvectors and *λ* a vector with the eigenvalues of Γ.

The power SNR for the k-th orthogonalized channel is then given by:

5 $$P{}_{k}=\frac{{\sigma }_{signal}^{2}{\lambda }_{k}}{\sum _{j}{({U}_{kj}{\sigma }_{noise})}^{2}}$$

From equation 5, it can be seen that the orthogonalization process mixes the sensor noise in the channels. As such, the noise in the channels will be partially correlated; the extent of this correlation is defined by the spatial properties of the lead-field matrix. The noise levels we used were σ_Noise,L*T*c_ = 3 fT/√Hz and σ_Noise,H*T*c_ = 50 (conservative) and 10 (optimistic) fT/√Hz. As described above, the source magnitude was physiologically constrained for each 0.6 mm^2^ cortical patch at σ_Signal_ = 0.4 nAm. *I*_*tot*_ then quantifies the total amount of information that is extracted from a single sample of the entire brain.

### Spatial information density maps

The spatial variation of the information capacity across the cortex provides insights into the coverage at the single-source level for different MEG arrays.

The SID value for a single patch/source in the brain is calculated as

6 $$SID{}_{patch}=\frac{1}{2}\sum _{k}{\rm{l}}{\rm{o}}{{\rm{g}}}_{2}(\frac{{\sigma }_{signal}^{2}{\lambda }_{k}}{\sum _{j}{({U}_{kj}{\sigma }_{noise})}^{2}}+1)$$

where *U*_*k*_ are the eigenvectors and *λ*_*k*_ are the eigenvalues of the matrix Γ_*patch*_ of the k-th orthogonalized channel. *SID*_*patch*_ is calculated by summing the independent information from all orthogonalized channels for that particular patch. Γ_*patch*_ for a single patch of the cortical surface is then:

7 $${{\rm{\Gamma }}}_{patch}={G}_{patch}{G}_{patch}^{T}$$

with ${G}_{patch}$ being the row vector of the gain matrix for a single source or patch.

The average of the SID values across the brain is given as

8 $$SI{D}_{avg}=\frac{1}{N}\sum _{n=1}^{N}SI{D}_{patch,n}$$

where N is the number of cortical patches/sources (156 176 and 127 931 for the adult and child, respectively). *SID*_*avg*_ then quantifies the average amount of information that is extracted via individually sampling all independent sources.

## Electronic supplementary material

Supplementary Information
