# Supplementary material for: Evaluation of realistic layouts for next generation on-scalp MEG: spatial information density maps
Source: Sci Rep. 2017 Aug 1;7:6974. doi: 10.1038/s41598-017-07046-6 (PMC5539206; doi:10.1038/s41598-017-07046-6)
Supplement: Supplementary file 1 — Supplementary Information [file 41598_2017_7046_MOESM1_ESM.doc]

**Supplementary Information**

**Evaluation of realistic layouts for next generation on-scalp MEG: spatial information density maps**

Bushra Riaz ^1^, Christoph Pfeiffer^2^, Justin F. Schneiderman^1*^

**Supplementary Figure 1:** Cumulative sum of information capacity for Elekta and high-*T*_c_ SQUID arrays as they sample the adult (left) and child (right) brains. The high-*T*_c_ SQUID noise level used here is 10 fT/√Hz whereas the Elekta SQUID noise is 3 fT/√Hz.
